# Advancing fat graft survival: from adipose-derived stem cell mechanisms to next-generation regenerative strategies

**DOI:** 10.3389/fcell.2026.1870729

**Published:** 2026-06-17

**Authors:** Xunuo Ma, Leiting Xu, Jianfei Zhang, Xiudi Wu, Tao Tao

**Affiliations:** 1 Zhejiang Wanli University, Ningbo, China; 2 Medical School of Ningbo University, Ningbo, China; 3 The First Affiliated Hospital of Ningbo University, Ningbo, China; 4 Ningbo College of Health Sciences, Ningbo, China

**Keywords:** adipose-derived stem/stromal cells, angiogenesis, cell-assisted lipotransfer, exosomal microRNA, fat graft survival, immunomodulation, mitochondrial transfer, oncological safety

## Abstract

Autologous fat grafting is widely used in reconstructive and aesthetic surgery because it is easy to harvest, well tolerated by the host, and produces natural-looking results. However, unpredictable graft resorption—with reported volume retention ranging from 30% to 80%—remains a major obstacle. To overcome this, two cell-based approaches have been developed: the stromal vascular fraction (SVF), a freshly isolated mixture containing approximately 1%–10% adipose-derived stem/stromal cells (ADSCs) plus other cell types, and culture-expanded ADSCs, a more homogeneous population obtained under Good Manufacturing Practice conditions. Meta-analyses show that while SVF increases fat retention by a modest margin, expanded ADSCs can achieve substantially greater improvements, highlighting important biological and regulatory differences between the two preparations. ADSCs support graft survival not only through classic growth factor secretion but also via immunomodulation, intercellular mitochondrial transfer, and exosomal microRNAs that co-ordinate angiogenesis, inflammation, and fibrosis. This review summarises current clinical evidence for ADSC- and SVF-enriched fat grafting in breast reconstruction, facial rejuvenation, scar management, chronic wounds, hair restoration, and paediatric as well as elderly populations. We also critically assess adjunctive strategies, including platelet-rich plasma, exosome-based acellular therapies, decellularized adipose matrices, engineered hydrogels, and nanofat grafting, and discuss the role of mitochondrial transfer enhancement. Standardised summary tables of clinical studies and emerging technologies are provided to facilitate evidence synthesis. Persistent challenges—such as the frequent conflation of SVF with ADSCs in the literature, the lack of standardised preparation and potency assays, and unresolved long-term oncological safety concerns—are examined, and priority areas for future research are proposed.

## 1 Why fat grafts fail—and how stem cells might rescue them

Autologous fat grafting is now a standard technique in both cosmetic and reconstructive surgery. Its popularity stems from three main advantages: the fat is easy to obtain, can be harvested repeatedly, and the procedure is relatively simple. The method was first described by Neuber in 1893 for correcting facial depressions, and since then it has moved beyond simple filling to become a tool for tissue replacement and regeneration (Hoyos et al., 2026). Today, autologous fat is widely used to repair tissue defects, treat post-radiotherapy changes, correct hemifacial or breast atrophy, and for various aesthetic purposes (Mohammed et al., 2026; Rigotti et al., 2016; Willemsen et al., 2014). Compared with synthetic fillers (e.g., silicone implants) or allogeneic materials, autologous fat is derived from the patient’s own body, so it provokes little immune reaction, leads to fewer post-operative complications such as rejection or foreign-body reactions, and gives a more natural look. A recent randomised controlled trial comparing breast implants with conventional lipofilling for breast hypoplasia confirmed that implants provide more pronounced and lasting volume, but lipofilling produces significantly more natural results and better correction of chest wall deformities like pectus excavatum (Gentile, 2026).

Nevertheless, the clinical use of autologous fat grafting is hampered by its unpredictable persistence. Resorption rates vary widely depending on the patient and the technique used (Bashir et al., 2023; Fu et al., 2022; Wang N. et al., 2025). A large systematic review covering 35 studies and 3,757 women who underwent breast augmentation with autologous fat grafting found an overall complication rate of 27.8%, with fat necrosis representing 43.7% of all complications, and an average volume retention of only 58% (range 44%–83%) (Seth et al., 2024). In large-volume fat grafting, ischaemia and poor nutrient supply often lead to fat necrosis, liquefaction, or cyst formation. As a result, multiple supplementary grafting sessions are often needed to maintain the volume, increasing the treatment burden on patients and limiting the wider use of autologous fat grafting in complex reconstructive surgery.

Advances in stem cell research have opened new possibilities. Stem cells can promote tissue repair, modulate inflammation, and improve the environment into which a graft is placed. In transplantation, adding stem cells has been shown to improve graft survival and functional integration (Chen et al., 2021). Building on this, two related but fundamentally different cell preparations have been explored to enhance autologous fat grafting: the stromal vascular fraction (SVF) and culture-expanded adipose-derived stem/stromal cells (ADSCs/ASCs). The approach is known as cell-assisted lipotransfer (CAL). It is essential to distinguish between SVF and ADSCs. SVF is a fresh, heterogeneous mixture of cells obtained from adipose tissue by enzymatic or mechanical processing. It contains about 1%–10% ADSCs together with endothelial cells, pericytes, fibroblasts, and immune cells, and can be used immediately without culture. In contrast, ADSCs are a relatively uniform population of mesenchymal stem/stromal cells purified from SVF by culturing and expanding them under Good Manufacturing Practice (GMP) conditions, typically reaching >90% purity. A recent meta-analysis of 31 studies showed that ex vivo-expanded ADSCs gave the largest improvement in fat retention (mean difference 64.6%; 95% CI: 60.5–68.7), whereas SVF produced moderate gains (mean difference 17.0%; 95% CI: 8.6–25.4) (Shimizu et al., 2026). This distinction has major regulatory implications: SVF is generally regarded as a minimally manipulated autologous tissue product, while cultured ADSCs are classified as an advanced therapy medicinal product (ATMP) requiring GMP facilities.

**FIGURE 1:**
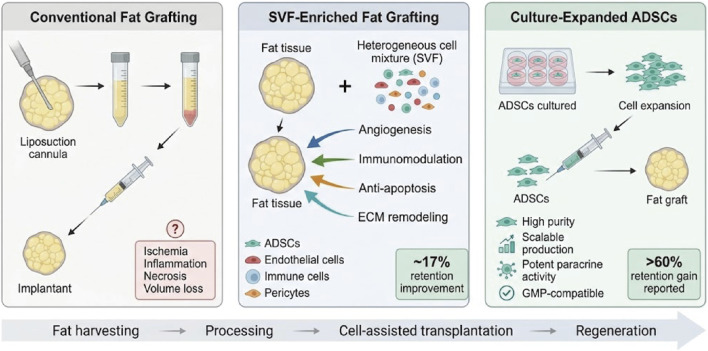
Comparative strategies of cell-assisted fat grafting. Schematic overview comparing conventional fat grafting, stromal vascular fraction (SVF)-assisted fat grafting, and culture-expanded adipose-derived stem cell (ADSC)-assisted fat grafting. Conventional fat grafting is limited by ischemia, inflammation, adipocyte necrosis, and unpredictable volume retention. SVF-assisted grafting introduces a heterogeneous cellular population containing ADSCs, endothelial cells, immune cells, and pericytes, which collectively promote angiogenesis, immunomodulation, anti-apoptotic effects, and extracellular matrix remodeling. In contrast, culture-expanded ADSCs provide a more homogeneous and scalable mesenchymal stromal cell population with enhanced paracrine activity and regenerative potential. These cell-assisted strategies improve vascular integration, reduce graft resorption, and enhance long-term fat graft retention.

ADSCs are a type of mesenchymal stem cell found in the SVF of adipose tissue. They are abundant, can be obtained with minimal invasiveness, have strong proliferative capacity, and can differentiate into multiple lineages. In CAL procedures, preparing cell-enriched fat grafts involves harvesting fat, then using either enzymatic digestion or mechanical processing, followed by centrifugation and mixing of the concentrated cells with purified fat. Enzymatic methods give higher purity but take more time and face stricter regulation; mechanical methods are simpler and more accessible clinically but yield fewer cells. A systematic review of 15 studies on mechanically processed adipose tissue reported that mechanical processing increased SVF cell density (in 87% of studies), improved extracellular matrix preservation (80%), raised expression of vascular markers such as CD31 and CD34 (67%), and reduced adverse histological features (oil cysts, fibrosis, inflammatory infiltrates) in 60% of studies (Schimanski et al., 2025). The isolated SVF is typically mixed with purified autologous fat at a defined ratio before being implanted (Figure 1). Compared with bone marrow-derived MSCs, ADSCs have practical advantages: they can be obtained in large numbers by low-trauma liposuction, yield more cells per volume of aspirate, and expand faster in culture while retaining multipotency (Jin et al., 2023; Mohamed-Ahmed et al., 2021).

In recent years, several ADSC-derived strategies have expanded the possibilities for adipose tissue transplantation. These include combining ADSCs with platelet-rich plasma (PRP), using cell-free ADSC exosomes, and employing bio-scaffolds, hydrogels, and other novel delivery systems. For example, PRP is rich in growth factors such as PDGF and VEGF, which activate the PI3K/AKT and ERK-MAPK pathways to promote endothelial cell proliferation and migration, accelerate neovascularisation, and facilitate preadipocyte differentiation via the p38/MAPK pathway. A recent study found that platelets can directly transfer functional mitochondria to ADSCs, restoring mitochondrial function and improving ADSC survival under oxidative stress during the early post-transplant period (Ke et al., 2025). Collectively, these effects enhance blood supply and cell survival in fat graft sites (Bensa et al., 2026; Qu et al., 2026; Perumal et al., 2023). ADSC-derived exosomes offer a cell-free option: they deliver molecular signals (mRNAs, miRNAs, proteins) to recipient cells, providing advantages in safety and quality control over live cell therapies (Hao, 2026; Jeong et al., 2024). Specific miRNAs carried by these exosomes have been identified as key mediators: miR-21-5p targets the NOTCH1/DLL4/VEGFA pathway in endothelial progenitor cells to promote angiogenesis (Cao et al., 2024); miR-125b-5p suppresses Smad2 to reduce hypertrophic scarring (Xu et al., 2024); and miR-204-5p targets TGF-β1 to promote scar-free healing of diabetic wounds (Song et al., 2025). Biomaterial scaffolds and hydrogels provide three-dimensional microenvironmental support for adipose tissue engineering, helping to overcome oxygen and nutrient limitations in large grafts (Smitterberg et al., 2026). Emerging technologies—such as decellularized adipose matrices, thermosensitive hydrogels loaded with ADSC exosomes, microfluidic-engineered microspheres for co-delivering ADSCs and growth factors, and nanofat grafting—have further enriched the translational toolkit. Promising future directions also include AI-assisted surgical planning and organ-on-a-chip platforms for preclinical testing (Soni and Abbott, 2026; Baptista et al., 2023; Lauschke and Hagberg, 2023; Giannakaki et al., 2025).

The aim of this review is to systematically present the roles of ADSCs and SVF in autologous fat transplantation and the optimisation strategies derived from them. We describe their biological characteristics, clinical applications, safety concerns, and emerging trends, providing a theoretical basis for further mechanistic research and clinical implementation. We critically evaluate the efficacy of these strategies in breast reconstruction and augmentation, facial rejuvenation, scar repair, chronic wound management, hair regeneration, hand rejuvenation, and in special populations (paediatric and elderly patients). We also analyse ADSC-derived approaches including PRP, exosomes and their non-coding RNA cargoes, decellularized adipose matrices, hydrogels, microsphere/nanotube systems, and nanofat. Throughout, we maintain a clear distinction between SVF and culture-expanded ADSCs, and we present standardised summary tables of clinical studies and emerging technologies to facilitate evidence synthesis and clinical translation. Finally, we discuss core challenges—standardised preparation and quality control, efficacy evaluation, long-term safety (including oncological considerations), and regulatory compliance—and propose research directions for clinical translation, aiming to establish more stable and verifiable technical pathways for regenerative repair.

## 2 The biology of fat-derived stem cells: more than just building blocks

### 2.1 Two products, two philosophies: why SVF and ADSCs cannot Be interchanged

Before examining how these cells work, we must first clarify what they are. In the field of fat grafting, two cell-based preparations are commonly used, but they are often confused: the stromal vascular fraction (SVF) and culture-expanded adipose-derived stem/stromal cells (ADSCs/ASCs). Although the literature frequently uses these terms interchangeably, they represent very different biological products with distinct cellular compositions, manufacturing processes, regulatory statuses, and clinical risk profiles.

SVF is a fresh, mixed population of cells isolated from lipoaspirate—either by enzymatic digestion with collagenase or by mechanical methods (filtration and centrifugation). Typically, SVF contains about 1%–10% ADSCs, plus endothelial cells, pericytes, smooth muscle cells, fibroblasts, macrophages, lymphocytes, and other blood-lineage cells. Because SVF is obtained without any *in vitro* culture and can be used immediately during surgery, most regulatory authorities classify it as a minimally manipulated autologous tissue product. In contrast, ADSCs are a much purer population. They are derived from SVF by allowing the cells to adhere to plastic culture surfaces and then expanding them *in vitro* under Good Manufacturing Practice (GMP) conditions. After expansion, they routinely achieve >90% purity, as assessed by surface markers (CD73^+^, CD90^+^, CD105^+^, CD45^−^, CD31^−^). This expansion takes 1 to 3 weeks and requires specialised facilities and strict quality control. Consequently, ADSCs are considered an advanced therapy medicinal product (ATMP) and are subject to much tighter regulation.

The clinical relevance of this distinction has been quantified. A high-quality systematic review and meta-analysis of 31 studies (1,426 patients) compared the two approaches. *Ex vivo*-expanded ADSCs improved fat retention by a large margin, whereas SVF gave only moderate gains (Shimizu et al., 2026). This difference is not just about cell purity; the secretome also differs. One study found that SVF secretes higher levels of tissue-remodelling cytokines (e.g., MMP-9, EMMPRIN, endoglin, IL-8) than either adipocyte grafts or cultured ADSCs, suggesting that the multicellular nature of SVF produces a more complex inflammatory and wound-healing profile (Carr et al., 2024). Another network meta-analysis of 31 trials showed that ADSC-assisted grafting achieved the highest graft survival rate and the lowest complication rate among all assisted methods, whereas SVF-assisted grafting had a higher complication rate (Dong et al., 2024).

Given these differences, we use standardised terminology throughout this review. “SVF-assisted lipotransfer” means the use of freshly isolated, uncultured SVF. “ADSC-assisted lipotransfer” (or simply CAL when the specific preparation is not specified or both are included) refers to the use of *in vitro* expanded ADSCs. It is worth noting that most published clinical studies to date have actually used SVF, not expanded ADSCs; we will indicate the specific preparation when discussing each study.

### 2.2 Where do these cells come from—and does it matter?

Human ADSCs are routinely isolated from autologous white adipose tissue, with the abdomen, buttocks, and inner thigh being the most common harvest sites. The ready availability, low-risk extraction, and minimal ethical concerns make them one of the most accessible mesenchymal stem cell sources (Farhana et al., 2026; Kahn et al., 2019). Compared with bone marrow-derived MSCs, ADSCs proliferate faster, adapt more easily to culture conditions, and can be expanded to clinically useful numbers without losing their multipotent properties (Mohamed-Ahmed et al., 2021).

However, donor-specific factors can influence ADSC behaviour. Donor age, for instance, affects SVF viability and ADSC proliferation and migration. One study found that younger donors (18–29 years) had better cell function than middle-aged (30–49 years) or elderly (50–65 years) donors, although adipogenic differentiation was preserved across all ages. Cryopreserved ADSCs from younger donors also improved fat graft survival more effectively in animal models (Qu et al., 2023). Yet another rigorous study reported no significant age-related differences in clonogenicity, trilineage differentiation, angiogenic growth factor secretion, or the ability to induce vascular sprouting in co-culture systems (Trotzier et al., 2024). This apparent contradiction highlights an important point: donor age alone is not a reliable predictor; individual functional testing of each cell product is needed.

Beyond age, the anatomical location of the fat matters. Superficial and deep subcutaneous adipose depots contain different ADSC subpopulations. Cells from the superficial layer have greater hyperplastic and angiogenic potential, whereas those from the deep layer express genes more typical of visceral fat, including a more inflammatory profile (Baptista et al., 2023). Processing technique also matters. A systematic review of mechanical processing methods found that they increased SVF cell density (87% of studies), improved ECM preservation (80%), raised vascular marker expression (CD31, CD34; 67% of studies), and reduced oil cysts, fibrosis, and inflammation (60% of studies) (Schimanski et al., 2025).

Finally, ADSCs are multipotent: under appropriate conditions they can become adipocytes, osteoblasts, chondrocytes, endothelial-like cells, neurite-like cells, or Schwann-like cells (Santoso et al., 2025). But their most clinically relevant role in fat grafting is not differentiation—it is their paracrine activity, which modulates angiogenesis, inflammation, extracellular matrix remodelling, and host cell recruitment.

### 2.3 Building new blood vessels: the first imperative for graft survival

A transplanted piece of fat faces an immediate crisis. For the first one to 3 days, no new capillaries have formed, so the tissue suffers from ischaemia, hypoxia, and lack of nutrients. ADSCs come to the rescue by secreting a battery of pro-angiogenic factors: VEGF-A, HGF, Ang-1, PlGF, bFGF, and others (Sadeghi et al., 2024; Furia et al., 2022; Badimon and Cubedo, 2017). These molecules bind to receptors (VEGFR2, c-Met, Tie2) and activate downstream pathways (PI3K/AKT, ERK/MAPK), leading to endothelial cell proliferation, migration, tube formation, and vessel stabilisation. Numerous animal studies have confirmed that ADSCs increase capillary density in grafted fat, improving oxygen and nutrient delivery while reducing fat cysts and fibrosis (Merhi, 2025). The pro-angiogenic effect is not a solo act; it involves crosstalk with smooth muscle cells, endothelial cells, and fibroblasts. Activation of PI3K-AKT and MAPK-ERK signalling by ADSCs has been shown to drive endothelial migration, tube formation, and tissue perfusion (Xiong et al., 2023). In recent years, the picture has grown more complex. ADSC-derived exosomes also carry pro-angiogenic miRNAs. For example, miR-21-5p activates the NOTCH1/DLL4/VEGFA axis in endothelial progenitor cells, promoting their proliferation, migration, and tube formation (Cao et al., 2024). Hypoxia-preconditioned ADSC exosomes loaded in GelMA hydrogels enhance type H angiogenesis via miR-21-5p targeting SPRY1 and activating PI3K/AKT (Li X. et al., 2024). Another exosomal miRNA, miR-100-5p, promotes angiogenesis while dampening inflammation by downregulating TNF-α and IL-6 in diabetic wound models (Liu H. et al., 2025). An interesting cautionary note: measuring only VEGF may not predict angiogenic potential. A study of 12 cultured ADSC samples found that two with high VEGF lacked co-expression of FGF19 and IGFBP-3, suggesting that a multi-factor profiling panel is needed for quality assessment (Teufelsbauer et al., 2025). In practice, optimising the fat-to-ADSC ratio (Chiu, 2019) and using layered, multi-point, low-pressure injection techniques can maximise the benefits (Lee et al., 2013; Chen et al., 2024).

### 2.4 Quieting the inflammatory storm

**FIGURE 2:**
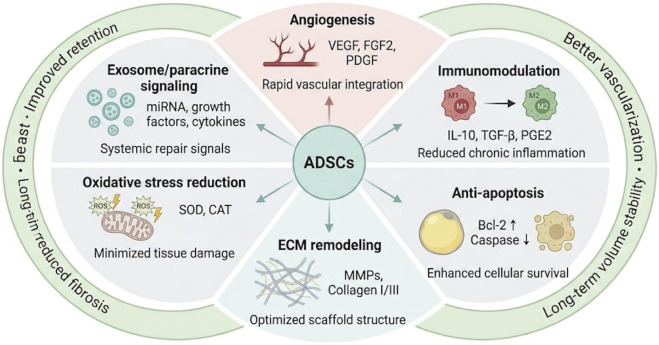
Mechanisms by which ADSCs enhance fat graft survival. Illustration of the major biological mechanisms through which adipose-derived stem cells (ADSCs) improve fat graft survival and tissue regeneration. ADSCs promote angiogenesis through secretion of vascular growth factors such as VEGF, FGF2, and PDGF, facilitating rapid vascular integration of transplanted tissue. ADSCs also modulate the immune microenvironment by promoting macrophage polarization toward an anti-inflammatory M2 phenotype and secreting immunoregulatory cytokines including IL-10 and TGF-β. Additional regenerative functions include inhibition of adipocyte apoptosis, reduction of oxidative stress, extracellular matrix remodeling, and paracrine/exosome-mediated signaling. Collectively, these mechanisms contribute to improved graft retention, reduced fibrosis, enhanced vascularization, and long-term volume stability.

ADSCs are also potent immunomodulators. After fat grafting, the tissue organises into three concentric zones: a necrotic core, an inflamed middle zone, and an outer regenerative layer (Saffari et al., 2022). ADSCs act within these zones to calm inflammation and promote repair. They secrete a mixture of anti-inflammatory factors: IL-10, TGF-β, PGE2, IDO, and TSG-6, which shift local immune cells from a pro-inflammatory to an anti-inflammatory, tissue-healing phenotype (Wu et al., 2026). A systematic review of radiation-induced fibrosis found that ADSC-treated tissues had more M2 (anti-inflammatory) macrophages, less interferon-γ signalling, and reduced fibroblast proliferation and collagen deposition (Pattani et al., 2024). ADSC paracrine activity also inhibits T-cell proliferation and helps balance CD4^+^ T-cell subsets at the wound site (Arya et al., 2026). During the inflammatory phase, ADSCs reduce natural killer cell function and stimulate T-cells to produce IL-10, thereby limiting post-operative inflammation (Tang et al., 2026). This is particularly valuable for chronic wounds, radiation fibrosis, and scar softening. A pressure ulcer study showed that ADSC exosomes carrying miR-21-5p induce M2 polarisation by inhibiting NF-κB, lowering TNF-α and IL-6 levels (Su Y. et al., 2025). Another exosomal miRNA, miR-125b-5p, suppresses Smad2 in hypertrophic scar fibroblasts, reducing collagen I and III deposition and preventing pathological scarring (Xu et al., 2024). Overall, ADSC-enriched fat grafts not only add volume but also improve immune balance, matrix remodelling, and long-term graft stability compared with conventional fat (Figure 2).

### 2.5 Mitochondria as a lifeline: a newly discovered survival circuit

One of the most exciting recent discoveries is that ADSCs can transfer functional mitochondria to stressed recipient cells via tunnelling nanotubes (TNTs). This intercellular mitochondrial transfer restores bioenergetic function under ischaemic and oxidative stress. Guo et al. showed that ADSCs transfer mitochondria to macrophages through TNTs, promoting M2 polarisation and improving fat graft retention (Guo et al., 2026). Zhang et al. found that thymosin β-4 pre-treatment enhances this transfer from ADSCs to adipocytes and endothelial cells via the Rac/F-actin pathway, reducing apoptosis and boosting vascularisation (Zhang X. et al., 2025). Even platelets can donate mitochondria to ADSCs under oxidative stress, restoring membrane potential and lowering ROS (Ke et al., 2025). This mitochondrial lifeline is a fundamental, targetable cytoprotective mechanism in cell-augmented fat grafting.

### 2.6 Weathering the ischaemic crisis: metabolic resilience and anti-death signals

ADSCs are inherently stress-resistant. They have low metabolic demands and strong antioxidant capacity under hypoxic conditions (Badimon and Cubedo, 2017), allowing them to survive the nutrient-deprived phase before new vessels form, while continuing to release beneficial cytokines. Thymosin β-4 (Tβ4) enhances this anti-apoptotic ability. *In vitro*, Tβ4 at certain concentrations increased ADSC proliferation, raised expression of angiogenesis-related genes, and modulated the Hippo signalling pathway (Li W. et al., 2024). Hypoxia preconditioning also helps: it upregulates pro-angiogenic factors (VEGF, PlGF) and downregulates anti-angiogenic ones (thrombospondin-1, PAI-1) (Li et al., 2023). This is especially relevant for elderly patients, whose native ADSCs may have reduced angiogenic capacity. These preconditioning strategies—hypoxia, Tβ4, mechanical stimulation—offer promising ways to boost ADSC potency before transplantation.

## 3 What the clinic tells us: evidence across anatomical sites and indications

### 3.1 The breast: where aesthetics, reconstruction, and oncological caution converge

Breast cancer incidence is rising, and more patients seek post-mastectomy reconstruction. Currently, silicone implants and autologous flaps are the main options. Implants offer shorter recovery and good early results, but carry risks of capsular contracture, infection, and foreign-body reactions, and their tolerance after radiotherapy is uncertain. Autologous flaps provide natural texture and stable volume, but cause donor site morbidity and prolonged recovery (Acharya et al., 2025; Giannas et al., 2026). A recent randomised trial comparing implants with conventional lipofilling for breast hypoplasia found that implants gave more pronounced volume augmentation, whereas lipofilling produced more natural results and better correction of chest wall deformities (Gentile, 2026). Against this background, autologous fat grafting has gained popularity because of its simplicity, ready availability, and natural appearance. A large systematic review of autologous fat grafting for breast augmentation established key benchmarks: complications include fat necrosis as the most common event; the average injected volume is in the range of a few hundred millilitres; and volume retention is modest but variable. Patient satisfaction at 1 year is generally high. Notably, adding PRP or SVF was associated with better retention (Seth et al., 2024). These data highlight both the utility of conventional fat grafting and the persistent problem of unpredictable volume loss that cell-augmented strategies aim to solve.

#### 3.1.1 Can stem cells make breasts bigger—and keep them that way?

The first clinical report of CAL for cosmetic breast augmentation described satisfactory volume and contour improvement, with only minor complications and no serious adverse events (Sun et al., 2025). A comparative study later reported substantially higher 1-year contour and volume retention in SVF-augmented grafts than in conventional lipofilling (Du et al., 2026). A subsequent follow-up study using MRI and biopsy found that SVF-enriched fat had markedly better vascularisation, correlating with improved volume retention. This confirmed that cellular enrichment promotes neovascularisation, reduces ischaemic damage, and lowers the incidence of fat cysts and calcifications (Acharya et al., 2025). Several meta-analyses have since quantified these benefits. A pooled analysis of multiple studies showed that SVF enrichment significantly improves fat retention compared with conventional grafting, although heterogeneity was high. Automated enzymatic isolation systems gave the largest retention gains, and the effect was larger in facial than in breast applications. Complication rates did not differ between enriched and conventional groups (Jefri et al., 2026). A subsequent meta-analysis of controlled trials confirmed better graft survival with enrichment, but extreme heterogeneity was again observed. An important insight from that analysis was the “harvest-quality ceiling” hypothesis: when water-assisted liposuction was used, cell enrichment added no benefit, whereas with standard aspiration, significant gains were seen. This suggests that a high-quality baseline harvest may make enrichment redundant (Sutar et al., 2026). Overall, SVF-CAL appears safe and effective for cosmetic breast augmentation, but its benefit is context-dependent.

#### 3.1.2 Rebuilding after cancer: a tougher test for fat grafts

Breast reconstruction after cancer surgery is more challenging. Post-mastectomy patients often have extensive tissue loss, poor blood supply, and radiation-induced fibrosis, all of which impair conventional fat graft survival. Enriched grafts can improve the local microenvironment by secreting angiogenic, immunomodulatory, and anti-fibrotic factors. A systematic review of radiation-induced fibrosis confirmed that fat transfer reduces fibroblast proliferation, collagen deposition, and pro-inflammatory signalling, while promoting M2 macrophage polarisation and extracellular matrix restoration. However, the effects on vascularisation were inconsistent (Pattani et al., 2024). A separate systematic review of CAL in irradiated patients found no increased recurrence risk and better aesthetics, but irradiated patients needed more fat and had lower retention than non-irradiated patients, indicating that the hostile recipient bed attenuates volumetric efficacy (Wu et al., 2023a). A prospective controlled trial in secondary breast reconstruction compared SVF-enriched fat with conventional fat. SVF enrichment significantly improved fat retention and produced better breast softness, less fat liquefaction, and fewer calcifications, with no difference in complications (Tissiani and Alonso, 2016). An innovative approach—the LIPO MULTI-SVF phase I/II trial—tested whether distributing SVF cells over multiple time points rather than as a single dose could improve persistence. SVF was given in three staggered aliquots. Short-term results showed increased graft thickness at 2 weeks that was maintained at 6 weeks, but thickness progressively declined by 6 months and 1 year. After several years of follow-up, no cancer relapses were reported, supporting safety (Agostini et al., 2025). The most robust evidence comes from a recent large-scale meta-analysis that stratified outcomes by preparation method. ADSC-enhanced grafting significantly improved fat retention compared with conventional grafting. *Ex vivo*-expanded ADSCs gave the greatest improvement, whereas SVF provided moderate gains. Complication rates were similar, and patient-reported outcomes favoured ADSC-enhanced grafting (Shimizu et al., 2026). This confirms that the preparation method—fresh SVF versus cultured ADSCs—modulates the magnitude of clinical benefit.

#### 3.1.3 The cancer question: safety data, preclinical warnings, and the unresolved tension

The oncological safety of autologous fat grafting in breast cancer patients has been intensely debated. Clinical studies have not shown that CAL increases local recurrence risk. The pooled analysis of the multicentre BREAST-I and BREAST-II trials compared patients who underwent autologous fat transfer for total breast reconstruction with matched controls from a cancer registry. After long-term follow-up, overall survival was not inferior in the fat transfer group, and after adjusting for confounders, fat transfer was not associated with increased mortality (van der Venne et al., 2026). A meta-analysis of patients with previous breast cancer found no significant difference in recurrence rates between ADSC-enhanced and conventional fat grafting (Shimizu et al., 2026). Another comprehensive meta-analysis covering both basic science and clinical studies concluded that autologous fat grafting does not increase breast cancer recurrence risk, with safety confirmed across various subgroups. However, the same analysis noted that basic science studies show ADSCs can promote breast cancer growth *in vitro* and in animal models, creating an unresolved tension between preclinical mechanisms and clinical outcomes (Wang et al., 2023). This tension is supported by mechanistic studies. One study showed that ADSCs promote proliferation and migration of premalignant breast epithelial cells and increase tumour incidence in a xenograft model. In a carcinogen-induced rat model, ADSCs enhanced tumour incidence, growth, and metastasis, although ADSCs alone could not induce tumours in normal tissue. Mechanistically, ADSC paracrine factors activated the oncogenic PI3K-AKT pathway, and blocking this pathway diminished the pro-tumour effects (Wu et al., 2025). Another RNA sequencing study found that even short-term co-culture of ADSCs with breast cancer cells induced transcriptomic shifts linked to aggressive traits and worse clinical outcomes (Vu et al., 2024). A systematic review of the basic science concluded that ADSCs exert a dual role: *in vitro*, they can influence cancer cell behaviour through paracrine signalling; *in vivo*, enriched fat grafting is generally safe and does not increase recurrence risk, but invasive carcinoma warrants special attention (Valente et al., 2024).

Taken together, the evidence supports the current clinical consensus: autologous fat grafting, including cell-enriched variants, does not increase breast cancer recurrence when performed in patients with complete tumour resection and low recurrence risk. Nevertheless, preclinical findings that ADSC factors can activate oncogenic pathways advocate a cautious, individualised approach. Preoperative risk screening, detailed informed consent, and extended follow-up are recommended. CAL in high-risk patients (e.g., BRCA mutations, incompletely resected tumours) requires careful consideration. Table 1 summarises key clinical studies of SVF- and ADSC-augmented fat grafting in breast reconstruction and augmentation.

**TABLE 1:** Summary of key clinical studies on SVF/ADSC-augmented fat grafting for breast applications.

Author and year	Study design	Sample size (n)	Application	Intervention	Control	Follow-up	Primary outcomes	Key findings
Yoshimura et al. (2008), Sun et al. (2025)	Prospective case series	40	Cosmetic breast augmentation	SVF-CAL	None (single-arm)	12 months	Volume retention, safety	Satisfactory volume and shape; no major complications
Gentile et al. (2012), Du et al. (2026)	Comparative cohort	33	Breast reconstruction	SVF-enriched AFG	Conventional AFG	12 months	3D volume retention	63% vs. 39% contour recovery favouring SVF
Tissiani and Alonso (2016)	Prospective controlled	24	Secondary breast reconstruction	SVF-enriched AFG	Conventional AFG	12 months	Fat retention rate	78.8% vs. 51.4% retention; comparable complications
Gentile et al. (2020), Acharya et al. (2025)	Retrospective cohort	50	Cosmetic breast augmentation	SVF-enriched AFG	Conventional AFG	12 months	MRI, tissue biopsy	Higher vascularisation in SVF group
Kølle et al. (2020), Merhi (2025)	RCT (triple-blind)	16	Cosmetic breast augmentation	ADSC-enriched AFG	Placebo (saline) AFG	12 months	MRI volume retention	ADSC group significantly superior volume retention
Agostini et al. (2025)	Single-arm clinical trial (phase I/II)	12	Post-mastectomy reconstruction	Staggered SVF-CAL (3 aliquots)	None (single-arm)	12 months (safety: 2.0–5.8 years)	Graft thickness, safety	Short-term improvement in thickness; long-term not sustained; no recurrence at 5.8 years
van der Venne et al. (2026)	Pooled multicentre RCT + nonrandomised trial	242 AFT vs. 19,936 controls	Total breast reconstruction	Conventional AFT	National registry controls	8.0 years (mean)	Overall survival	10-year OS 97.6% vs. 75.7%; HR 0.34 (P = 0.025)
Shimizu et al. (2026) (Meta)	Systematic review and meta-analysis (31 studies)	1,426 (634 ADSC, 792 conventional)	Breast reconstruction	ADSC- or SVF-enhanced AFG	Conventional AFG	Variable	Fat retention, complications, recurrence	MD 26.8% retention; 64.6% (expanded ADSCs) vs. 17.0% (SVF); RR 1.07 for complications; RR 1.56 for recurrence
Jefri et al. (2026) (Meta)	Systematic review and meta-analysis (18 studies)	893	Breast and facial	SVF-enriched AFG	Conventional AFG	Variable	Fat retention, complications	MD 17.20% retention; 22.8% with automated enzymatic systems; RR 1.15 for cysts/necrosis
Dong et al. (2024) (NMA)	Network meta-analysis (31 trials)	1,656	Multiple sites	ADSC-, SVF-, PRP-, SM-assisted AFG	Simple AFG	Variable	Graft survival, complications	ADSC highest survival (SUCRA 82.17%) and lowest complications (SUCRA 41.00%); SVF higher complications (SUCRA 65.14%)

SVF, stromal vascular fraction; CAL, cell-assisted lipotransfer; ADSC, adipose-derived stem/stromal cell; AFG, autologous fat grafting; RCT, randomised controlled trial; MD, mean difference; RR, risk ratio; SUCRA, surface under the cumulative ranking curve; NMA, network meta-analysis; OS, overall survival; HR, hazard ratio.

### 3.2 The face: volume, skin quality, and the pursuit of natural rejuvenation

Facial ageing involves fat atrophy, volume loss, and skin laxity. Traditional rejuvenation options—facelifts, hyaluronic acid, collagen, and conventional fat grafting—each have limitations. Facelifts are traumatic; fillers are temporary; fat grafting often needs repetition due to resorption. To address these, SVF- and ADSC-enriched fat grafting has been incorporated into facial protocols. Enriched grafts enhance vascularisation and improve the dermal microenvironment through secreted factors, increasing fat survival while improving skin elasticity and radiance (He et al., 2019; García-Contreras et al., 2014). A mini-review concluded that ADSC-enriched grafts increase collagen, elastin, and CD31 levels, reduce the need for repeat procedures, and benefit patients with fibrotic facial deformities (Ansari Lari et al., 2024).

#### 3.2.1 Does cell enrichment actually improve facial fat grafting?

A dedicated systematic review and meta-analysis of facial filling studies compared CAL with conventional lipotransfer. Fat survival and patient satisfaction were significantly higher with CAL, while complication rates were similar, and secondary operations were fewer (Shen et al., 2024). Initial studies used nanofat together with ADSCs injected into the superficial dermis. This increased skin thickness, reduced wrinkles, and raised hydration at 6 months, without infections, cysts, or granulomas (Jones and Toriumi, 2026). Later comparative trials found that SVF-augmented fat grafting gave smoother, more natural facial contours at 1 year, along with better skin elasticity and hydration (La Padula et al., 2023). A split-face randomised trial comparing SVF-enriched fat with conventional fat in the temples showed that the SVF group had higher graft survival, more natural contour, and better skin texture (Roshdy et al., 2022). Collectively, these studies show that SVF enrichment not only enhances fat survival but also improves skin quality. Recent innovations have focused on layer-specific processing. A retrospective study of multilayer facial fat grafting injected high-density fat and adipose matrix into deep compartments and SVF-gel into the periorbital region. Patient satisfaction improved substantially, though some patients required secondary procedures due to partial absorption (Yang et al., 2026). The pyramidal multi-type, multi-method, multi-layer (3M) strategy—injecting macrofat, microfat, and nanofat or SVF into distinct lower eyelid planes—significantly improved tear trough deformities, eye bags, and dark circles. Mild adverse effects (oedema, ecchymosis) were rare, and no major complications (including vision loss) occurred (Zhu et al., 2026).

#### 3.2.2 Acne scars: a surprising success story for SVF

Cell-enriched therapies have shown promise for atrophic acne scars. A systematic review and meta-analysis of autologous cell-based therapies (SVF, ReCell®, fat grafting) found a significant reduction in scar severity scores, with the largest effect for SVF. Patient satisfaction was higher, objective scar depth was reduced, and wound healing was accelerated. Adverse events were less frequent in the intervention groups (Zhang et al., 2026). Another systematic review specifically compared fat grafting, PRP, and SVF for acne scars. SVF had the highest rate of excellent improvement, followed by fat grafting and then PRP. Moderate and mild improvement rates followed a different order. Notably, SVF had no post-inflammatory hyperpigmentation or haematoma, whereas PRP carried some risk. Pain was most common with fat grafting and lower with PRP (Han et al., 2023). Thus, SVF may offer the best efficacy-to-safety ratio for this indication.

#### 3.2.3 From dark circles to facial paralysis: expanding the indications

For structural infraorbital dark circles, a study combined deep ligament-releasing micro-fat injection with superficial SVF-gel. At follow-up, dark circles and tear trough deformities improved, and patient satisfaction was high. This shows that cell-augmented facial fat grafting is not only for age-related volume loss but also for congenital or structural periorbital concerns in young adults (Su Y. S. et al., 2025). In facial paralysis reconstruction, ADSCs have shown neuroregenerative potential. A review highlighted that ADSCs can differentiate into Schwann-like cells, support axonal regeneration, and release neurotrophic factors. While not yet equivalent to nerve autografts, ADSC-enriched fat grafting offers an alternative for patients with facial nerve paralysis (Santoso et al., 2025).

#### 3.2.4 When things go wrong: vascular complications in facial grafting

Facial fat grafting carries unique anatomical risks. The periorbital region and nasal root are high-risk areas where intravascular injection can cause ophthalmic artery occlusion and blindness. A case report of severe skin necrosis after augmentation rhinoplasty underscored the need for high-precision imaging, fine-gauge tools, and thorough anatomical knowledge (Jiang et al., 2024). Table 2 summarises key clinical studies of SVF/ADSC-augmented fat grafting in facial rejuvenation and related applications. Despite positive results, personalised treatment remains underutilised. Protocols tailored to age, skin condition, and facial structure—such as the multilayer and 3M strategies—are important steps. Integrating PRP, biomaterial scaffolds, or other therapies may further improve outcomes. Safety vigilance is essential.

**TABLE 2:** Summary of key clinical studies on SVF/ADSC-augmented fat grafting for facial applications.

Author and year	Study design	Sample size (n)	Application	Intervention	Control	Follow-up	Primary outcomes	Key findings
Tonnard et al., (2013), Jones and Toriumi (2026)	Prospective case series	67	Superficial facial wrinkles, scars, periorbital	Nanofat grafting	None (single-arm)	6 months	Clinical improvement, safety	Improved skin quality; no severe complications
Bielli et al. (2014), La Padula et al. (2023)	Comparative cohort	23	Facial scars	SVF-enriched AFG + PRP	Conventional AFG	12 months	Volume retention, skin elasticity	∼66% vs. ∼39% fat survival favouring SVF
Roshdy et al. (2022)	Split-face randomised	15	Temporal augmentation	SVF-enriched AFG (one side)	Conventional AFG (contralateral)	6 months	Ultrasound biomicroscopy volume	Significantly higher survival in SVF side; improved skin texture
Shen et al. (2024), Shen et al. (2024) (Meta)	Systematic review and meta-analysis (15 studies)	737	Facial filling	CAL (SVF or ADSC)	Conventional lipotransfer	Variable	Fat survival rate, satisfaction, complications	SMD 3.04 for survival; RR 1.34 for satisfaction; RR 0.52 for secondary operation
Zhang et al. (2026) (Meta)	Systematic review and meta-analysis (18 studies)	500	Atrophic acne scars	SVF, ReCell®, fat grafting	Saline, laser alone	Variable	ECCA score, satisfaction, scar depth	SMD −1.25 ECCA; SMD −1.40 for SVF subgroup; RR 1.45 satisfaction
Han et al. (2023) (Meta)	Systematic review and meta-analysis	—	Acne scars	SVF vs. PRP vs. fat grafting	Various	Variable	Goodman and Baron scale, safety	SVF excellent improvement 73% vs. fat 36% vs. PRP 0%; SVF zero pigmentation/haematoma
Su Y. S. et al., (2025)	Prospective case series	88	Structural infraorbital dark circles	Micro-fat (deep) + SVF-gel (superficial)	None (single-arm)	11.2 months	Patient satisfaction, complication rate	97.7% satisfaction; no major complications
Yang et al. (2026)	Retrospective study	105	Multilayer facial rejuvenation	HDF, AMC, SVF-gel (layered)	None (single-arm)	Variable	GAIS, satisfaction (VAS)	Satisfaction 5.26→8.01; GAIS 1.88; 15.2% required secondary procedure
Zhu et al. (2026)	Retrospective study	53 (106 sides)	Infraorbital aging (3M strategy)	Macrofat, microfat/SEFF, nanofat/SVF (layered)	None (single-arm)	15.5 months (median)	Modified Goldberg scale, SSA scale	Significant improvement on both scales; 3.8% mild oedema/ecchymosis; 22 sides required revision

SVF, stromal vascular fraction; ADSC, adipose-derived stem/stromal cell; CAL, cell-assisted lipotransfer; AFG, autologous fat grafting; PRP, platelet-rich plasma; HDF, high-density fat; AMC, adipose matrix complex; SEFF, superficial enhanced fluid fat; 3M, multi-type, multi-method, multi-layer; ECCA, Echelle d'Évaluation Clinique des Cicatrices d'Acné; GAIS, global aesthetic improvement scale; VAS, visual analog scale; SSA, subject satisfaction assessment; SMD, standardised mean difference; RR, risk ratio.

### 3.3 Scars and wounds: turning fibrosis into regeneration

Scarring and chronic wounds share post-traumatic inflammation-fibrosis imbalance and local hypoperfusion, creating an unfavourable recipient environment for fat grafts (Foti et al., 2026). Traditional methods (excision, skin grafts, topical agents) have limitations, especially after radiotherapy or in extensive fibrosis. SVF- and ADSC-enriched fat grafting shifts the approach from pure volume replacement to microenvironment remodelling, optimising inflammation, blood supply, and matrix conditions to enhance adipocyte survival.

#### 3.3.1 Softening the irreversible: fat grafting against scar tissue

Cell-augmented fat grafting helps scar management through both volume support and microenvironment regulation. ADSC-secreted factors (TGF-β, MMPs, IL-10) inhibit overactive fibroblasts, reduce excessive collagen deposition, and remodel extracellular matrix (Wang L. et al., 2025; Nseir et al., 2017). A comprehensive review noted that ADSCs secrete pro-angiogenic and neurotrophic factors, exert immunomodulatory effects, and contribute to neoangiogenesis, reduced pain and itching, and fibrosis attenuation (Ahmad et al., 2024). Mechanistically, ADSC exosomes directly target the fibrotic cascade. One study showed that ADSC exosomes accelerate wound healing, improve quality, and prevent pathological scar formation by inhibiting myofibroblast transdifferentiation and downregulating fibrosis markers. miR-125b-5p in exosomes suppresses Smad2, and exosome administration alleviated skin fibrosis in an animal model (Xu et al., 2024). A systematic review of fat grafting for burn scars found that all included studies reported postoperative scar improvement. When validated scales were used, the improvement exceeded the minimal clinically important difference. The authors recommended standardised scales for future research (El Sewify et al., 2025). Another systematic review of fat grafting and ADSC therapy for acute burns and scars confirmed improvements in wound healing, vascularisation, and scar characteristics, though functional gains were minimal (Lesmanawati et al., 2024). A prospective study combining hybrid fractional laser with autologous lipofilling for scar remodelling showed greater improvement in pigmentation, elasticity, pliability, and thickness compared with laser alone. Pain and itching were also better alleviated, attributed to the regenerative properties of ADSCs and SVF (Delia et al., 2025). In an understudied application, autologous fat grafting was evaluated in survivors of female genital mutilation with vulvar scars. Significant improvements were seen in vulvar aesthetics, genital self-image, sexual function, and psychological wellbeing (Almadori et al., 2025).

#### 3.3.2 Healing the unhealable: chronic wounds as a test bed

Standard care for chronic wounds (debridement, dressings, metabolic management) often yields suboptimal closure rates. ADSCs and SVF have been introduced as microenvironment remodellers. A prospective study of non-healing wounds of various aetiologies treated with emulsified autologous fat grafting found that most wounds healed without additional intervention, with a mean time to complete healing of several weeks (Prakash et al., 2024). Two randomised controlled trials of SVF in chronic wounds showed that autologous extracellular matrix/SVF gel significantly enhanced healing rates compared with standard dressings (Deng et al., 2018), and that SVF cell therapy accelerated healing, improved final closure rates, and reduced time to complete healing (Carstens et al., 2021). However, a prospective observational study added nuance: PRP alone achieved substantial healing, and adding autologous adipose tissue transfer did not confer extra benefit, suggesting that the incremental value of cell enrichment depends on wound characteristics (Riza et al., 2025).

In diabetic foot ulcers, debridement plus autologous fat transplantation led to a substantial mean reduction in ulcer area by 4 weeks, with most wounds closing within about 2 months (Yin et al., 2021). A randomised trial comparing fat alone, fat plus PRP, and conventional care found that the fat-plus-PRP group had higher microvascular density at 1 week and better histological appearance, indicating that even in diabetic patients, cell-enriched signals accelerate revascularisation (Mo et al., 2023). Preclinical studies have elucidated exosomal miRNA mechanisms. Hypoxia-preconditioned ADSC exosomes carrying miR-100-5p enhance endothelial and fibroblast function *in vitro* and reduce ulcer size, increase VEGF, and decrease inflammation in a rat DFU model (Liu H. et al., 2025). ADSC exosomes also promote fibroblast proliferation and migration while reducing excessive myofibroblast differentiation via miR-204-5p targeting TGF-β1, promoting scar-free healing (Song et al., 2025). Exosomes from miR-21-5p-overexpressing ADSCs promote wound healing and reduce inflammation in a pressure ulcer model by inducing M2 polarisation (Su Y. et al., 2025).

In Martorell hypertensive leg ulcers, a pilot study of autologous fat grafting reported accelerated healing, pain relief, and prevention of recurrence (Rodríguez Franco et al., 2026). Collectively, ADSC- and SVF-enriched therapies improve the tissue microenvironment and angiogenesis across different chronic wound settings. Table 3 summarises key clinical studies for scar repair and chronic wound management.

**TABLE 3:** Summary of key clinical studies on SVF/ADSC-augmented fat grafting for scar repair and chronic wound management.

Author and year	Study design	Sample size (n)	Application	Intervention	Control	Follow-up	Primary outcomes	Key findings
Scar repair								
El Sewify et al. (2025)	Systematic review (14 studies)	885	Burn scars	AFG ± ADSCs	Variable	Variable	POSAS, VSS, clinical assessment	Mean POSAS improvement 7.28 (MCID < 1); 100% of studies reported improvement
Delia et al. (2025)	Prospective comparative	—	Cutaneous scars (various)	Hybrid laser + lipofilling	Hybrid laser alone	90 days	VSS, VAS, satisfaction	Combined therapy significantly superior for pigmentation, elasticity, pliability, thickness, pain, itching
Almadori et al. (2025)	Prospective case series	13	FGM vulvar scars	Autologous fat grafting	None (single-arm)	12.23 months	VASS, FGSIS, FSFI, HADS	Significant improvement in all scales; QoL enhancement
Xu et al. (2024) (Preclinical)	*In vitro* + *in vivo* (mouse)	—	Hypertrophic scarring	ADSC-Exos (miR-125b-5p/Smad2)	PBS control	Variable	Fibrosis markers, histology	Accelerated healing + prevented scar formation; inhibited α-SMA, COL1, COL3
Lesmanawati et al. (2024)	Systematic review (6 studies)	3–100	Acute burns and burn scars	AFG ± ADSCs	Variable	Variable	Wound healing, vascularisation, scar characteristics	Most studies reported improvement; functional outcomes minimal; limited acute burn evidence
Chronic Wound Management								
Deng et al. (2018)	STROBE-compliant study	—	Chronic wounds	ECM/SVF gel	Standard dressings	Variable	Wound closure rate	Significantly enhanced healing with SVF gel
Tanios et al. (2021), Carstens et al. (2021)	RCT	—	Chronic ulcers	SVF cells	Standard care	Variable	Healing rate, closure rate	Significant acceleration of healing; improved final closure
Stasch et al. (2015), Yin et al. (2021)	Prospective case series	—	Diabetic foot ulcers (refractory)	Debridement + autologous lipotransfer	None (single-arm)	68 days	Wound closure rate	88% complete closure; 50% area reduction at 4 weeks
Nolan et al. (2022), Mo et al. (2023)	RCT	18	Diabetic foot ulcers	AFG + PRP vs. AFG alone vs. conventional	Three-arm comparison	—	Microvascular density, histology	Fat+PRP group: 1,645 ± 96 vessels/mm^2^; significantly higher angiogenesis
Prakash et al. (2024)	Prospective study	18	Non-healing wounds (various)	Emulsified AFG	Baseline (conventional dressing × 2 weeks)	5.05 weeks (mean)	Wound healing rate	95% complete healing; mean healing time 5.05 weeks
Riza et al. (2025)	Prospective observational	31	Chronic wounds (various)	PRP ± autologous adipose tissue	Various	Variable	Wound area reduction	>80% area reduction in 64%; complete healing 48.3%; fat grafting did not confer additional benefit over PRP alone
Liu H. et al. (2025) (Preclinical)	*In vitro* + *in vivo* (rat DFU)	—	Diabetic foot ulcers	Hypoxic ADSC-Exos (miR-100-5p)	PBS control	Variable	Ulcer size, VEGF/CD31, TNF-α/IL-6	Reduced ulcer size; increased angiogenesis; decreased inflammation
Song et al. (2025) (Preclinical)	*In vitro* + *in vivo* (diabetic wound)	—	Diabetic wounds (scar-free healing)	ADSC-Exos (miR-204-5p/TGF-β1)	PBS control	Variable	Fibrosis markers, histology	Enhanced fibroblast function; reduced myofibroblast differentiation and collagen deposition
Su Y. et al. (2025) (Preclinical)	*In vitro* + *in vivo* (mouse pressure ulcer)	—	Pressure ulcers	ADSC-Exos (miR-21-5p/NF-κB)	PBS control	Variable	Wound closure, M2 polarisation, TNF-α, IL-6	Accelerated healing; M2 polarisation via NF-κB inhibition

SVF, stromal vascular fraction; ADSC, adipose-derived stem/stromal cell; AFG, autologous fat grafting; PRP, platelet-rich plasma; DFU, diabetic foot ulcer; POSAS, patient and observer scar assessment scale; VSS, vancouver scar scale; VAS, visual analog scale; VASS, vulvar architecture severity scale; FGSIS, Female Genital Self-Image Scale; FSFI, female sexual function index; HADS, hospital anxiety and depression scale; Exos, exosomes; ECM, extracellular matrix; MCID, minimal clinically important difference; QoL, quality of life.

#### 3.3.3 What the evidence still cannot tell us

Several limitations persist. First, most studies are small case series or retrospective analyses; few RCTs exist, and those have modest sample sizes. Second, cellular preparations are heterogeneous—most use whole adipose tissue or mechanically isolated SVF, not cultured ADSCs. Third, outcome assessment tools vary widely, impeding meta-analysis. Fourth, follow-up is generally short, inadequate for assessing durability of scar improvement or long-term wound stability. Fifth, the distinction between physical volume effect and biological activity of cellular constituents has not been systematically disentangled. Well-designed, multicentre RCTs with standardised preparations, core outcome sets, and extended follow-up are urgently needed.

### 3.4 Broadening the scope: other indications, special populations, and evidence limitations

#### 3.4.1 Beyond the usual targets: hair, hands, and other Frontiers

SVF- and ADSC-enriched fat grafting is no longer limited to the face and breast. Its applications now span hair restoration, hand rejuvenation, gluteal augmentation, and head-and-neck reconstruction. Across these diverse sites, the cellular fraction of the graft improves the local microenvironment through the same angiogenic, immunomodulatory, and trophic mechanisms outlined in Chapter 2, thereby supporting graft survival and tissue integration.

##### 3.4.1.1 Hair restoration

Androgenetic alopecia (AGA) involves progressive follicular miniaturisation driven by androgen signalling, and conventional therapies often yield limited results. ADSCs can prolong the anagen phase of hair follicles by secreting growth factors such as FGF, VEGF, and EGF (Coriddi et al., 2022; Liu Q. et al., 2025). A systematic review of 20 clinical studies covering five regenerative modalities (conditioned media, platelet-rich fibrin, SVF, extracellular vesicles, and stem cells) found that SVF performed comparably or better than platelet-rich plasma in terms of hair density, and that extracellular vesicles and stem cell therapies also showed positive effects. Only mild, transient adverse events were reported (Behrangi et al., 2026). A prospective study of SVF injection in AGA patients documented improvements in hair shaft calibre, hair count, and the terminal-to-vellus ratio, with no sex-related differences in response (El-Khalawany et al., 2023). Mechanistic studies suggest that oxidative stress can enhance the protective capacity of ADSCs via Nrf2-dependent pathways (Sun and Chen, 2025). Regenerative therapies—particularly those based on SVF and ADSCs—offer a promising option for AGA with a favourable safety profile, though larger, standardised trials are needed.

##### 3.4.1.2 Hand rejuvenation

Ageing hands show subcutaneous fat atrophy, dermal thinning, and prominent tendons and veins. Autologous fat grafting provides durable volume and the regenerative benefits of its cellular constituents. A systematic review of 11 studies reported consistently high patient satisfaction and low complication rates. The long-term retention and regenerative effects of ADSCs—such as promotion of collagen and elastin synthesis and anti-inflammatory microcirculatory modulation—are considered key contributors to success (Abu Alqam et al., 2025). Contemporary studies with objective volumetric tools are still needed, but cell-enriched fat grafting already stands as an attractive alternative to temporary fillers for hand rejuvenation (Debuc et al., 2023).

##### 3.4.1.3 Gluteal augmentation

In gluteal augmentation, the large volumes of fat grafted make the angiogenic and adipogenic support from SVF or ADSCs particularly valuable, as graft thickness directly correlates with the risk of central ischaemia and necrosis. Although clinical evidence is less abundant than for the breast or face, the biological rationale supports continued investigation and cautious clinical adoption of cell-augmented strategies in body contouring (Elsaftawy et al., 2025).

##### 3.4.1.4 Head and neck cancer reconstruction

An emerging application is soft-tissue restoration in head and neck cancer patients. A scoping review found no evidence of increased recurrence or metastasis after autologous fat grafting in this population, and favourable functional and aesthetic outcomes were consistently reported. However, the authors called for standardised protocols and extended follow-up to confirm safety (Correas et al., 2026). This underscores the versatility of cell-augmented fat grafting as a reconstructive tool across different oncological settings. Table 4 summarises key studies of SVF/ADSC-augmented fat grafting in these and other aesthetic/reconstructive applications.

**TABLE 4:** Summary of key studies on SVF/ADSC-augmented fat grafting in other aesthetic and reconstructive applications.

Author and year	Study design	Sample size (n)	Application	Intervention	Control	Follow-up	Primary outcomes	Key findings
Hair regeneration								
El-Khalawany et al., (2023)	Prospective study	30	AGA	Single-session autologous SVF injection	None (single-arm)	6 months	Hair shaft calibre, hair count/cm^2^, terminal/vellus ratio	Calibre: 0.037→0.056 mm; count: 130.87→151.93/cm^2^; T/V ratio: 77.06→81.45%
Behrangi et al. (2026) (SR)	Systematic review (20 studies)	724	AGA	CM, PRF, SVF, EV, SCs	Various	Variable	Hair density, hair count, safety	SVF: 41%–48% density increase; EV: 28% count increase; SCs: 30% density in 2 months; all with mild transient AEs
Sun and Chen (2025) (Preclinical)	*In vitro* + *in vivo* (mouse)	—	Alopecia (radiation-induced)	H_2_O_2_-pretreated ADSC-CM	Non-pretreated ADSC-CM	Variable	Hair regrowth, Nrf2 activation, oxidative markers	Pretreated ADSC-CM superior; Nrf2-dependent paracrine mechanism
Hand rejuvenation								
Abu Alqam et al. (2025) (SR)	Systematic review (11 studies)	303	Hand rejuvenation	Autologous fat transfer	Variable (3 RCTs, 7 case series, 1 prospective)	5–38 months	Patient satisfaction, complications, volume retention	Consistently high satisfaction; infrequent/minor complications
Head and neck reconstruction								
Correas et al. (2026) (ScR)	Scoping review (5 studies)	116 (largest series)	Head and neck cancer reconstruction	Autologous fat grafting	Variable	Variable	Oncological safety, functional/aesthetic outcomes	No evidence of increased recurrence; favourable contour and tissue quality
Gluteal augmentation								
Condé-Green et al. (2016), Elsaftawy et al. (2025)	Systematic review and meta-analysis	—	Gluteal augmentation	AFG ± cell enrichment	Variable	Variable	Volume retention, complications	Cell enrichment improved retention and reduced complications

SVF, stromal vascular fraction; ADSC, adipose-derived stem/stromal cell; AGA, androgenetic alopecia; CM, conditioned media; PRF, platelet-rich fibrin; EV, extracellular vesicles; SCs, stem cells; AFG, autologous fat grafting; SR, systematic review; ScR, scoping review; AE, adverse event; T/V ratio, terminal-to-vellus hair ratio.

#### 3.4.2 One size does not Fit all: Special populations, special considerations

The efficacy of cell-augmented fat grafting varies across paediatric, elderly, and chronically ill patients, because both the quality of the cellular product and the receptivity of the host tissue are influenced by age and disease.

##### 3.4.2.1 Children and adolescents

Paediatric patients require smaller fat volumes and more meticulous techniques. Low-pressure micro-aspiration and closed mechanical enrichment systems help preserve cellular viability and reduce contamination (Kaufman et al., 2007). Paediatric-derived mesenchymal stem cells show higher proliferative capacity and longer telomeres than adult cells (Zomer et al., 2026), and children have more brown/beige adipose tissue, which may influence ADSC properties (Nagarajan et al., 2025). In children, ADSC-augmented fat grafting is primarily used for functional restoration and correction of congenital anomalies rather than age-related volume loss. For example, ultrasound-guided micro-fragmented fat injection into the anal sphincter complex improved faecal incontinence in paediatric patients, with increased sphincter thickness and no major complications (Batista et al., 2026). In a 16-year-old with bladder exstrophy, stem cell-augmented fat grafting increased abdominal wall thickness substantially at 3 months with good cosmetic results (Li et al., 2026). A multicentre randomised trial demonstrated that autologous SVF and fat grafting promote skin regeneration during tissue expansion, a technique often used in paediatric reconstruction. Both treatments significantly increased skin thickness and the expansion index at 12 weeks, with no severe adverse events during 2 years of follow-up (Tan et al., 2026). Another retrospective study in congenital muscular torticollis found that percutaneous myotomies combined with enriched fat grafting produced excellent or good functional and aesthetic improvement in the majority of patients, with no relapses (Monreal, 2025). Thus, the small-volume, function-oriented strategy in children capitalises on the heightened regenerative potential of paediatric tissues.

##### 3.4.2.2 Elderly patients

Aging affects native ADSCs, reducing their proliferation, migration, and angiogenic capacity while increasing senescence-associated and pro-inflammatory signals (Chen et al., 2016; Griffin et al., 2017). The effect of donor age on ADSC function is debated: one study found age-related declines in SVF viability and cell performance (Qu et al., 2023), whereas another reported no difference in clonogenicity, differentiation, or angiogenic capacity between young and old donors (Trotzier et al., 2024). The key lesson is that functional characterisation of individual cell products is more important than chronological age. Strategies to counteract age-related decline include hypoxic preconditioning, which restores the pro-angiogenic factor expression of elderly ADSCs (Shi et al., 2024), and thymosin β-4, which promotes mitochondrial transfer and reduces oxidative stress (Ding et al., 2025). In elderly patients, fat graft survival is generally lower than in younger adults. Nevertheless, a clinical histological study showed that ADSC intervention improved skin elasticity, vascular density, and wrinkle depth, with clinically meaningful volume retention (Wang et al., 2017). A comprehensive review concluded that ASC-mediated modulation of inflammation, fibrosis, and vascularisation can partially compensate for age-related recipient bed deficiencies (Trotzier et al., 2023).

##### 3.4.2.3 Patients with chronic systemic disease

Chronic conditions such as diabetes and hypertension create a hostile recipient environment characterised by persistent inflammation, microvascular insufficiency, and immune dysregulation. In systemic sclerosis, SVF-enriched fat grafting has shown promise in reducing cutaneous and digital manifestations; most studies report positive outcomes and no life-threatening adverse events (Jafarzadeh et al., 2026). Another systematic review noted that SVF therapy reduced digital ulcers and pain in scleroderma patients, while ADRC-assisted grafting provided limited benefit in breast cancer-related lymphoedema (Hakami et al., 2025). However, a randomised trial in breast cancer-related lymphoedema found no clinical effect of ADRC-assisted lipotransfer, and single-cell RNA sequencing revealed no differences in cell composition or gene expression between responders and non-responders. The authors recommended against routine use of this approach (And et al., 2024). This negative result highlights the need for rigorous patient selection and the recognition that not all chronic conditions respond equally. Future research must optimise cell preparation and delivery strategies specifically for adverse host environments.

#### 3.4.3 A critical look at the clinical evidence: what holds us back

Despite generally positive findings, several limitations must be acknowledged. First, most studies are small case series, retrospective analyses, or single-arm prospective cohorts; adequately powered randomised controlled trials are scarce. The lack of large, multicentre RCTs is the single biggest gap. Second, the heterogeneity of cellular preparations is substantial. SVF and culture-expanded ADSCs are often conflated, yet they differ fundamentally in composition and biological activity. Moreover, isolation methods (enzymatic vs. mechanical), cell doses, and administration protocols vary widely. Meta-analyses confirm that the magnitude of benefit differs between SVF and expanded ADSCs, and automated systems yield different results than manual methods (Shimizu et al., 2026; Jefri et al., 2026; Dong et al., 2024). Standardisation of cell products and reporting is urgently needed. Third, outcome assessment tools are inconsistent across studies—ranging from subjective evaluations to validated scales and advanced imaging. This impedes meta-analysis. Core outcome sets should be developed for each application domain. Fourth, follow-up durations are generally short (6–12 months), insufficient to assess long-term volume stability or oncological safety. Notable exceptions, such as the BREAST-I/II trials with extended follow-up, demonstrate the value of long-term surveillance (van der Venne et al., 2026). Fifth, publication bias may skew the literature toward positive results; the high heterogeneity observed in meta-analyses suggests that negative or neutral findings remain unpublished. A few studies showing no added benefit of cell enrichment (Sutar et al., 2026; Riza et al., 2025) are important correctives. Finally, clinical studies have not systematically disentangled the physical volume effect of grafted fat from the specific biological contributions of its cellular fraction. Controlled trials comparing volume-matched enriched and non-enriched grafts are needed.

### 3.5 Patient-reported outcomes and quality of life

While objective measures such as volume retention, complication rates, and imaging-based assessments dominate the literature on cell-augmented fat grafting, the patient’s own perception of treatment success is equally critical. Patient-reported outcome measures (PROMs) capture domains that clinical metrics cannot: aesthetic satisfaction, psychological wellbeing, social functioning, and symptom relief (e.g., pain, itching, or tightness). In breast reconstruction, for example, a patient may accept modest volume loss if the natural feel and contour are maintained, whereas another may be dissatisfied despite excellent volumetric retention due to asymmetry or scarring. PROMs therefore provide a more holistic evaluation of clinical benefit.

Unfortunately, PROMs are inconsistently used in studies of SVF- and ADSC-enhanced fat grafting. Many trials report global satisfaction rates without validated instruments, making cross-study comparisons unreliable. Few have employed standardised tools such as the BREAST-Q for breast surgery, the FACE-Q for facial procedures, or the Patient and Observer Scar Assessment Scale (POSAS) for scar outcomes. The BREAST-Q, for instance, includes domains for satisfaction with breasts, psychosocial wellbeing, sexual wellbeing, and physical chest symptoms. Its use in cell-assisted lipotransfer trials would allow meaningful benchmarking against implant-based or flap-based reconstruction. Similarly, in facial rejuvenation, validated scales like the FACE-Q satisfaction with facial appearance and aging appraisal could quantify whether cell enrichment offers added value beyond conventional fat grafting.

The importance of PROMs extends to special populations. For paediatric patients undergoing functional reconstruction (e.g., for faecal incontinence), child- and parent-reported outcome tools are essential to capture quality-of-life improvements that may not correlate with anatomical changes. In elderly patients, PROMs can assess whether improved skin quality and reduced wrinkle depth translate into meaningful daily benefits. Moreover, in chronic wound management, patient-reported pain, sleep disturbance, and mobility are often as important as wound closure rates.

Another under-utilized outcome is the psychological impact of scarring and disfigurement. Studies reviewed in Section 3.3 have shown that fat grafting can improve scar pliability, colour, and thickness, but whether these changes alleviate patient distress or improve social confidence remains largely unquantified. One notable exception is the study on female genital mutilation survivors (Almadori et al., 2025), which included validated measures of genital self-image, sexual function, and psychological wellbeing, demonstrating that fat grafting can have profound quality-of-life effects beyond aesthetic improvement.

Going forward, we recommend that all clinical trials of cell-augmented fat grafting incorporate at least one disease-specific or procedure-specific PROM as a secondary endpoint. Core outcome sets for each indication should be developed by international consensus, including both objective volumetric measures and validated patient-reported domains. Only by combining these two perspectives can we truly determine whether the additional complexity and cost of cell enrichment are justified from the patient’s standpoint. Furthermore, future research should explore the minimal clinically important difference (MCID) for PROMs in this field, enabling sample size calculations and meaningful interpretation of trial results.

## 4 Beyond whole cells: the next-generation of regenerative strategies

In addition to using whole ADSCs or SVF, researchers have developed several adjunctive and cell-free strategies to improve fat graft survival. These include platelet-rich plasma (PRP), ADSC-derived exosomes, and various biomaterial scaffolds. Each approach aims to support the graft during the vulnerable early post-transplantation period by enhancing vascularisation, modulating inflammation, or providing structural support.

### 4.1 Platelet-rich plasma (PRP) as an adjunct

PRP is produced by centrifuging autologous blood to concentrate platelets. Its therapeutic effect comes from the growth factors stored in platelet α-granules, which can stimulate endothelial cells and promote matrix remodelling (Hasiba-Pappas et al., 2022). When used together with SVF or ADSCs, PRP is intended to improve the survival and function of the transplanted cells by creating a more favourable local environment (Elsharkawi et al., 2025). Key factors released from PRP—such as PDGF, VEGF, and TGF-β—support early blood vessel formation (Ruhl et al., 2019). A recent systematic review identified PRP and ADSC-based methods as the most promising enhancements for fat grafting (Hasiba-Pappas et al., 2025).

Beyond growth factor release, a recent study uncovered a novel mechanism: platelets can transfer functional mitochondria to ADSCs under oxidative stress, reducing reactive oxygen species and improving cell viability. In an animal model, this mitochondrial transfer enhanced graft vascularisation and long-term retention (Ke et al., 2025).

Clinical studies on PRP-augmented fat grafting have shown mixed but generally positive results. In a trial of breast cancer patients undergoing breast-conserving surgery, PRP assistance reduced the number of injection sessions and lowered complication rates, with higher patient satisfaction (Kirch, 2025). For traumatic scars, combining PRP-enriched fat grafting with non-ablative laser therapy accelerated healing and produced better scar appearance than either treatment alone (Lynch and Bashir, 2016).

However, the benefit of adding PRP is not universal. In a cohort of chronic wound patients, PRP alone achieved good healing rates, and the additional use of autologous fat transfer did not further improve outcomes (Riza et al., 2025). A network meta-analysis comparing different assisted grafting techniques found that PRP ranked below ADSC-assisted grafting in terms of graft survival and was associated with a higher complication rate (Dong et al., 2024). These findings suggest that PRP is not always superior to cell-based strategies; its value depends on the specific clinical indication, the cell-to-PRP ratio, and patient selection.

### 4.2 Exosomes from ADSCs: cell-free messengers

ADSCs release small extracellular vesicles (30–200 nm), known as exosomes, which carry miRNAs, lncRNAs, circRNAs, proteins, and lipids. These vesicles mediate intercellular communication and have attracted interest as a cell-free alternative to stem cell therapy (Alfarafisa et al., 2025). A review focusing on non-coding RNAs in ADSC exosomes highlighted their roles in angiogenesis, oxidative stress reduction, inflammation modulation, skin regeneration, and scar prevention (Rong et al., 2025). Because exosomes lack live cells, they avoid risks such as immune rejection, tumourigenicity, and aberrant differentiation, and are easier to store and standardise (Gong et al., 2025; Liang et al., 2025).

Preclinical studies have elucidated several pathways by which ADSC exosomes improve fat graft survival. They promote angiogenesis through miRNAs such as miR-21-5p (targeting NOTCH1/DLL4 and SPRY1/PI3K/AKT) (Cao et al., 2024; Li X. et al., 2024) and miR-100-5p (enhancing vessels while suppressing inflammation) (Liu H. et al., 2025). They also support preadipocyte differentiation via Wnt/β-catenin and PPARγ modulation (Zhang J. et al., 2025; Tang et al., 2025; Si et al., 2025; Su H. et al., 2025; Tian et al., 2025; Jin and Zhou, 2026). Immunomodulation is achieved through M2 macrophage polarisation (miR-21-5p/NF-κB) (Su Y. et al., 2025) and inhibition of inflammasome activation (Gong et al., 2025; Liang et al., 2025). Anti-fibrotic effects are mediated by miR-125b-5p targeting Smad2 (Xu et al., 2024) and miR-204-5p targeting TGF-β1 (Song et al., 2025). Additionally, exosomes upregulate antioxidant pathways (SIRT3/SOD2) and protect mitochondrial function in ischaemia-reperfusion settings (Zhao et al., 2026; Azimian Zavareh et al., 2026; Wang B. et al., 2025; Lou et al., 2025a; Lou et al., 2025b; Lou et al., 2024a). In animal models of fat grafting, exosome treatment accelerated neovascularisation via the HIF-1α/VEGF axis while suppressing Notch/DLL4, and simultaneously shifted the balance from Wnt/β-catenin toward PPARγ, resulting in better fat integrity and retention than control (Tang et al., 2025). For delivery, hydrogels such as PF-127 or GelMA have been used to encapsulate exosomes, achieving sustained release and improved outcomes (Li X. et al., 2024; Liu E. et al., 2025). It is important to note that all current evidence on ADSC exosomes for fat grafting comes from preclinical studies. No clinical trial has yet tested their efficacy in humans. Major hurdles remain, including standardisation of isolation and characterisation, development of potency assays, long-term safety validation, and scalable GMP-compliant production (Huang et al., 2025).

### 4.3 Engineering a better graft with biomaterials

Biomaterial scaffolds are designed to provide a supportive framework for transplanted cells and adipose tissue. They serve two main functions: offering physical support during the ischaemic phase and enabling controlled release of pro-regenerative signals. This section covers decellularized matrices, hydrogels, and micro-/nanoscale carriers.

#### 4.3.1 Decellularized adipose matrix (DAM)

DAM is produced by removing cellular components and lipids from adipose tissue while preserving the extracellular matrix (collagens, glycosaminoglycans, and adhesion proteins) (Ikezaki et al., 2024). The resulting scaffold can bind and slowly release growth factors such as VEGF, FGF, and TGF-β, and it promotes host cell infiltration, angiogenesis, and adipogenesis after transplantation (Gorecka et al., 2018; Wang et al., 2018). The anatomical origin of the adipose tissue influences DAM properties: material from superficial fat depots contains more type I collagen and larger pores, which better support ADSC adipogenic differentiation than deep-fat-derived DAM (Derby et al., 2014). In preclinical studies, DAM or autologous adipose tissue (AAT) scaffolds have shown good biocompatibility and volume retention in mouse models. Co-transplantation with ADSCs further enhanced new fat formation and vascularisation (Dong et al., 2025; Shoham et al., 2013). Clinical case reports have documented the safety and feasibility of allogeneic DAM products (e.g., Renova) for soft tissue augmentation, with low inflammation and good integration (Baniameri et al., 2025; Kim et al., 2025). Although these studies did not include ADSCs, they suggest that combining DAM with ADSCs could offer additional benefits for graft survival and long-term volume maintenance.

#### 4.3.2 Hydrogels

Hydrogels are three-dimensional, water-rich networks that mimic the extracellular matrix and are injectable. They provide physical support during the early ischaemic period, maintaining graft shape and allowing nutrient diffusion (Tan et al., 2017; Lee et al., 2018). When loaded with cytokines or exosomes, hydrogels can sustainably release pro-angiogenic signals, delaying apoptosis and improving vascularisation (Liu E. et al., 2025). Preclinical experiments have shown that encapsulating adipose tissue in a pectin-alginate hydrogel increased vascularisation and cell survival, with imaging and histology confirming enhanced angiogenesis and adipogenesis (Wu et al., 2023b). Similarly, PF-127 thermosensitive hydrogels loaded with ADSC exosomes improved graft volume and weight compared to controls (Liu E. et al., 2025), and GelMA hydrogels with hypoxia-preconditioned exosomes stimulated type H angiogenesis via SPRY1/PI3K/AKT signalling (Li X. et al., 2024). A clinical feasibility study using a hyaluronic acid-based hydrogel to deliver ADSCs demonstrated good biocompatibility and volume retention trends, with no serious adverse reactions, providing early support for this approach in humans (Teixeira and Martins, 2023).

#### 4.3.3 Micro-/nanoscale carriers and nanofat

Advanced bioengineering has produced micron- and nanoscale delivery systems. For example, PLGA microspheres co-encapsulating VEGF and Ang-1, when implanted with ADSCs, increased graft volume in mouse models (Yin et al., 2023). Microfluidic-engineered porous GelMA microspheres carrying both ADSCs and FGF19 enhanced angiogenesis in an ischaemic limb model (Wu et al., 2023c). The most clinically advanced micronisation strategy is nanofat—emulsified adipose tissue that can be injected through fine needles. Originally described by Tonnard and colleagues, nanofat grafting was shown to improve skin quality in superficial indications such as fine lines, scars, and lower eyelid pigmentation, without major complications. Subsequent studies have combined nanofat with fractional CO_2_ laser for atrophic acne scars and with PRP or hair transplantation for alopecia, with encouraging but preliminary results. Future work should focus on standardising nanofat preparation, exploring combination therapies, and expanding indications (Jones and Toriumi, 2026; Meglei et al., 2024; Qin et al., 2025). Table 5 summarises the emerging ADSC-derived technologies discussed in this chapter, including their proposed mechanisms, advantages, limitations, and current evidence status.

**TABLE 5:** Summary of emerging ADSC-derived technologies for enhancing fat grafting outcomes.

Technology	Mechanism of action	Key advantages	Limitations and challenges	Preclinical evidence	Clinical evidence in plastic surgery
PRP + ADSC/SVF	Growth factor synergy (PDGF, VEGF, TGF-β); platelet-to-ADSC mitochondrial transfer (Ke et al., 2025)	Autologous, readily available, low cost, complementary angiogenic support	Heterogeneous preparation protocols; higher complication rate than ADSC alone in NMA (Dong et al., 2024)	Extensive (multiple animal models)	Moderate (breast reconstruction, scar repair, chronic wounds)
ADSC-Exos	miRNA-mediated angiogenesis (miR-21-5p/NOTCH1/DLL4, miR-877); immunomodulation (miR-21-5p/NF-κB/M2); anti-fibrosis (miR-125b-5p/Smad2, miR-204-5p/TGF-β1) (Cao et al., 2024; Xu et al., 2024; Song et al., 2025; Su Y. et al., 2025)	Cell-free, low immunogenicity, stable storage, avoids live-cell risks	No clinical trials published; standardisation of isolation/dosing undefined; GMP manufacturing challenges	Extensive (multiple animal models of fat grafting, wound healing, bone repair)	None (entirely preclinical)
Decellularized Adipose Matrix (DAM)	ECM scaffold providing structural support and growth factor binding; induces host cell infiltration and angiogenesis	Tissue-specific biochemical signals, low immunogenicity, off-the-shelf potential	Variable preparation methods; source-dependent properties; limited clinical data with ADSC co-delivery	Moderate (nude mouse models with ADSCs)	Limited (case reports of DAM alone for soft tissue augmentation)
Hydrogels (GelMA, PF-127, HA, pectin-alginate)	3D structural support during ischaemic phase; controlled release of exosomes/growth factors; injectable	Tunable degradation, versatile loading capacity, minimally invasive delivery	Long-term degradation products; variable *in vivo* performance; limited human data	Extensive (fat graft models with exosome/growth factor loading)	Very limited (HA-ADSC construct exploratory study)
Microspheres (PLGA, GelMA)	Sustained release of VEGF/Ang-1; co-delivery of ADSCs and growth factors	Injectable, biodegradable, high surface-area-to-volume ratio	Complex fabrication; regulatory hurdles for combination products	Moderate (mouse fat graft and ischaemic limb models)	None
Nanofat	Mechanical emulsification producing ADSC- and growth factor-rich injectable; superficial dermal regeneration	Simple preparation, no enzymes, clinically accessible	Limited volumetric capacity; mechanism relies on endogenous SVF	Limited	Moderate (facial rejuvenation, acne scars, alopecia)
Mitochondrial Transfer (TNT-mediated)	ADSC-to-macrophage FAO↑ and M2 polarisation (Guo et al., 2026); ADSC-to-adipocyte/endothelial cell energy rescue (Ding et al., 2025); platelet-to-ADSC mitochondrial cytoprotection (Ke et al., 2025)	Addresses bioenergetic crisis directly; targetable via Tβ4, Rac/F-actin pathway	Mechanistically novel; clinical translatability not yet demonstrated; TNT modulation *in vivo* challenging	Growing (mouse fat graft and wound models)	None

PRP, platelet-rich plasma; ADSC, adipose-derived stem/stromal cell; SVF, stromal vascular fraction; Exos, exosomes; DAM, decellularized adipose matrix; GelMA, gelatin methacryloyl; PF-127, Pluronic F-127; HA, hyaluronic acid; PLGA, poly(lactic-co-glycolic acid); TNT, tunnelling nanotube; NMA, network meta-analysis; FAO, fatty acid β-oxidation; GMP, good manufacturing practice.

## 5 The roadblocks and the road ahead

Despite the promise of ADSC-augmented fat grafting, several interrelated challenges limit its translation into routine clinical practice. This section discusses these obstacles and proposes directions for future research.

### 5.1 Lack of standardisation in cell manufacturing

A major bottleneck is the absence of validated, uniform protocols for producing ADSCs. Many variables—donor age, harvest technique, digestion method, and culture conditions—lead to wide batch-to-batch variability in cell yield, viability, and potency (Avila-Portillo et al., 2020). Moreover, no consensus exists on optimal cell dosing, with reported concentrations spanning orders of magnitude across studies, making direct comparisons difficult (Paganelli et al., 2022). Meta-analyses confirm that automated enzymatic isolation systems outperform manual methods in retention gains (Jefri et al., 2026), and that expanded ADSCs give greater benefits than fresh SVF (Shimizu et al., 2026). Yet, no widely adopted standard protocol exists. To overcome this, researchers have called for closed automated systems or rigorously controlled manual protocols with in-process checks (Avila-Portillo et al., 2020). Uniform criteria for cell characterisation—such as specific surface markers (e.g., CD73, CD90, CD105, CD45, CD31), viability thresholds, and secretome profiles—are essential (Jia et al., 2023). Developing reference standards and potency assays, similar to those used for other advanced therapy medicinal products, would greatly improve regulatory compliance and cross-study comparability.

### 5.2 Inconsistent outcome assessment

A second barrier is the lack of standardised, validated efficacy measures. As noted in Section 3.6, outcome assessment tools vary widely: breast reconstruction studies use everything from subjective evaluation to 3D imaging and MRI; facial rejuvenation studies employ 2D photography, 3D volumetry, ultrasound, or patient-reported scales; scar studies sometimes use validated scales (POSAS, VSS) but often do not; chronic wound research uses disparate metrics like closure rate, healing time, or area reduction. This heterogeneity impedes meta-analysis. The adoption of international core outcome sets for each indication would greatly enhance the value of future trials.

### 5.3 Safety concerns, especially oncological

Safety issues continue to temper enthusiasm. On the oncological front, long-term follow-up studies (including the BREAST-I/II trials) have not shown increased breast cancer recurrence after autologous fat grafting, whether conventional or cell-enriched (van der Venne et al., 2026; Wang et al., 2023). However, preclinical work has raised legitimate concerns: ADSC-secreted factors can activate the PI3K–AKT pathway in premalignant breast cells (Wu et al., 2025), and even short co-culture can induce transcriptomic shifts associated with aggressive cancer phenotypes (Vu et al., 2024). Therefore, clinical consensus recommends CAL only for patients with complete tumour resection, low recurrence risk, and an adequate disease-free interval, with full informed consent and extended follow-up. For head and neck cancers, a scoping review found no increased recurrence, but acknowledged limited evidence quality (Correas et al., 2026). Regarding immunological safety, autologous ADSCs and SVF have low rejection risk. Allogeneic ADSCs, which might be needed for patients with poor autologous cell function, could provoke immune responses (Ko et al., 2023; Kamprom et al., 2024; Technau et al., 2011). Rare but catastrophic adverse events—such as blindness after intravitreal injection of SVF—underscore the need for anatomical expertise and proper patient selection (Lou et al., 2024b; Dibiase et al., 2026; Dong et al., 2026; Juguilon et al., 2025). The long-term safety of repeated SVF or ADSC administrations, and the potential for cumulative genomic changes in cultured cells, remain unaddressed.

### 5.4 Regulatory and ethical complexities

Global regulatory frameworks vary widely. In the US, SVF is generally treated as a minimally manipulated autologous product (HCT/P) under 21 CFR 1271, while cultured ADSCs are regulated as drugs/devices requiring an IND. In the EU, both SVF and expanded ADSCs fall under the ATMP regulation, demanding GMP manufacturing and marketing authorisation. These differences hinder international trials and commercial development (Avila-Portillo et al., 2020). Ethical issues include donor privacy, informed consent, transparency about experimental nature, and the proliferation of unlicensed “stem cell clinics”. Potential solutions include gene editing (CRISPR/Cas) to remove pro-tumour or immunogenic factors from ADSCs, combined with 3D bioprinting to create immune-privileged constructs. Harmonised international guidelines, developed through bodies like the ICH, are urgently needed to regulate the entire chain from donor screening to clinical application, preventing misuse by unqualified practitioners.

### 5.5 What the next decade might bring: emerging technologies

**FIGURE 3:**
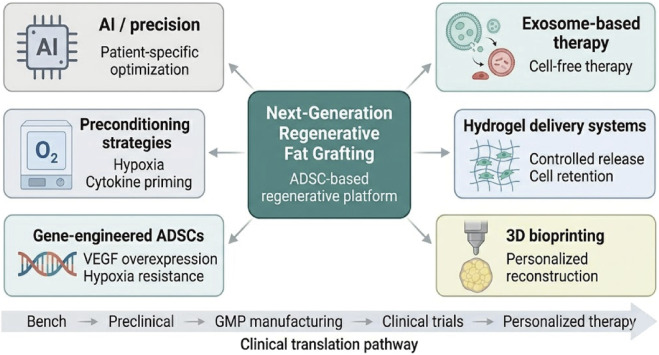
Next-generation regenerative strategies for fat grafting. Overview of emerging next-generation regenerative strategies designed to improve the efficacy and clinical translation of ADSC-based fat grafting. Advanced approaches include exosome-based cell-free therapy, hydrogel-based delivery systems for enhanced cell retention and controlled release, three-dimensional (3D) bioprinting for personalized tissue reconstruction, gene-engineered ADSCs with enhanced angiogenic or hypoxia-resistant properties, preconditioning strategies such as hypoxic or cytokine priming, and artificial intelligence (AI)-assisted precision medicine for patient-specific optimization. The lower panel illustrates the translational pathway from bench research and preclinical studies to GMP manufacturing, clinical trials, and personalized regenerative therapy.

Several cutting-edge innovations offer hope for overcoming the above barriers. AI-assisted planning can optimise injection points, depths, and volumes based on patient-specific anatomy, and predictive models are being developed for fat graft retention and personalised reconstruction (Giannakaki et al., 2025; Dibiase et al., 2026). Organ-on-a-chip and advanced 3D culture platforms enable controlled study of ADSC–host interactions, with potential applications in potency testing and personalised therapy (Soni and Abbott, 2026; Baptista et al., 2023; Lauschke and Hagberg, 2023). Single-cell, spatial, and multi-omics analyses will elucidate the molecular determinants of graft survival, identify the relevant cell subpopulations, and help develop potency assays that link molecular signatures to clinical outcomes. CRISPR-engineered ADSCs with enhanced regenerative activity, as well as preconditioning strategies (hypoxia, thymosin β-4, mitochondrial enhancement), represent promising next-generation products. In summary, ADSC-assisted fat grafting is moving from empirical practice toward a more standardised, intelligent, mechanism-driven, and personalised era. Realising this vision will require sustained collaboration between surgeons, cell biologists, engineers, regulators, and health economists. The most urgent priority remains well-designed, adequately powered, multicentre RCTs using standardised cell products, core outcome sets, and long-term follow-up (Figure 3).

### 5.6 Multi-centre registries and interdisciplinary collaboration

The heterogeneity of current evidence—spanning different cell preparations, isolation methods, injection techniques, and outcome measures—has hindered the widespread adoption of cell-augmented fat grafting. As discussed in Sections 5.1–5.4, standardisation of manufacturing, outcome assessment, and regulatory pathways is urgently needed. However, even with standardised protocols, the long-term safety and efficacy of SVF and ADSC grafting can only be reliably established through large-scale, prospective, multi-centre registries. These registries capture real-world data across diverse patient populations, surgical settings, and follow-up durations, complementing the controlled but often artificially restricted environment of randomised trials.

A well-designed registry should collect at least the following core variables: donor demographics (age, BMI, comorbidities), harvest and processing method (enzymatic vs. mechanical, cell dose, viability), injection technique (volume, layering, recipient site), concurrent treatments (e.g., PRP, laser, radiotherapy), and standardised outcomes (volume retention by 3D imaging, complication rates, and PROMs). Crucially, for oncological applications, registries must include long-term cancer surveillance (minimum 5–10 years) to definitively exclude recurrence risk. The BREAST-I/II trials (van der Venne et al., 2026) demonstrated the feasibility of linking fat grafting data to cancer registries; scaling this approach through multi-centre collaboration would provide definitive safety evidence.

Moreover, registries enable subgroup analyses that individual trials cannot power—such as the effect of donor age, radiation history, or specific comorbidities. They can also identify best practices by comparing outcomes between centres that use different protocols, facilitating iterative quality improvement. For emerging technologies (exosomes, hydrogels, nanofat), registries can track adoption patterns and early complication signals before large randomised trials are feasible.

Realising the potential of registries requires interdisciplinary collaboration. Plastic surgeons must agree on minimum data sets and adopt standardised terminology (e.g., clear distinction between SVF and ADSCs). Cell biologists and manufacturing experts must provide assays that can be performed in routine clinical laboratories to characterise cell products. Regulatory scientists and health economists should be involved in designing registry endpoints that satisfy payer and regulator requirements. Data scientists and biostatisticians are needed to manage longitudinal data and adjust for confounding.

Importantly, registries should be designed from the outset as “learning health systems” that can adapt as new evidence emerges. For example, when a new preconditioning strategy (e.g., hypoxic culture) shows promise in preclinical studies, a registry could monitor its real-world effectiveness and safety after controlled introduction. Similarly, as AI-assisted injection planning (Giannakaki et al., 2025; Dibiase et al., 2026) becomes available, registries can validate whether it improves retention or reduces complications.

In summary, we call for the establishment of an international, open-access registry for cell-augmented fat grafting, funded by professional societies (e.g., ISAPS, ASPS) and supported by industry partners. Interdisciplinary working groups should define core variables, data governance, and funding models. Only through such collaborative efforts can we generate the high-quality, long-term evidence needed to move cell-augmented fat grafting from an experimental innovation to an accepted standard of care in regenerative plastic surgery.

## 6 Where we stand and where we need to go

Both SVF and culture-expanded ADSCs improve fat graft survival through a combination of angiogenic, immunomodulatory, anti-fibrotic, and mitochondrial transfer mechanisms. Most clinical evidence—derived predominantly from SVF—shows consistent gains in volume retention for breast, facial, and scar applications, without evidence of increased cancer recurrence. Adjunctive and cell-free strategies (PRP, exosomal miRNAs, biomaterial carriers) offer additional microenvironmental benefits, though most are still preclinical. Critical obstacles include the lack of standardised cell preparation protocols, the persistent conflation of SVF with ADSCs in the literature, and insufficient long-term safety data. Overcoming these obstacles through harmonised standards, rigorous randomised trials, and emerging technologies will be essential to establish cell-augmented fat grafting as an evidence-based pillar of regenerative plastic surgery.
